# Serum Levels of Copper and Zinc and Survival in Breast Cancer Patients

**DOI:** 10.3390/nu16071000

**Published:** 2024-03-29

**Authors:** Marek Szwiec, Wojciech Marciniak, Róża Derkacz, Tomasz Huzarski, Jacek Gronwald, Cezary Cybulski, Tadeusz Dębniak, Anna Jakubowska, Marcin R. Lener, Michał Falco, Józef Kładny, Piotr Baszuk, Joanne Kotsopoulos, Steven A. Narod, Jan Lubiński

**Affiliations:** 1 Department of Surgery and Oncology, University of Zielona Góra, Zyty 28, 65-046 Zielona Góra, Poland; szwiec72@gmail.com; 2 Read-Gene, Grzepnica, ul. Alabastrowa 8, 72-003 Dobra, Poland; wojciech.marciniak@read-gene.com (W.M.); roza.derkacz@read-gene.com (R.D.); 3 Department of Clinical Genetics and Pathology, University of Zielona Góra, ul. Zyty 28, 65-046 Zielona Góra, Poland; tomasz.huzarski@pum.edu.pl; 4 Department of Genetics and Pathology, International Hereditary Cancer Center, Pomeranian Medical University in Szczecin, ul. Unii Lubelskiej 1, 71-252 Szczecin, Poland; jacek.gronwald@pum.edu.pl (J.G.); cezarycy@pum.edu.pl (C.C.); tadeusz.debniak@pum.edu.pl (T.D.); anna.jakubowska@pum.edu.pl (A.J.); marcin.lener@pum.edu.pl (M.R.L.); piotr.baszuk@pum.edu.pl (P.B.); 5 Regional Oncology Centre, 71-730 Szczecin, Poland; falco.miw@op.pl; 6 Department of General and Oncological Surgery, Pomeranian Medical University, 71-252 Szczecin, Poland; jkladny@onet.pl; 7 Women’s College Research Institute, Toronto, ON M5S 1B2, Canada; joanne.kotsopoulos@wchospital.ca (J.K.); steven.narod@wchospital.ca (S.A.N.); 8 Dalla Lana School of Public Health, University of Toronto, Toronto, ON M5T 3M7, Canada

**Keywords:** copper, breast cancer, survival, zinc, copper/zinc ratio

## Abstract

There is emerging interest in the relationship between several serum micronutrients and the prognosis of patients with breast cancer. The relationship between serum zinc and copper levels and breast cancer prognosis is unclear. In our study, we included 583 patients with breast cancer diagnosed between 2008 and 2015 in the region of Szczecin, Poland. In a blood sample obtained before treatment, serum zinc and copper levels were quantified by mass spectroscopy. Each patient was assigned to one of four categories (quartiles) based on the distribution of the elements in the entire cohort. Patients were followed from diagnosis to death over a mean of 10.0 years. The 10-year overall survival was 58.3% for women in the highest and 82.1% for those in the lowest quartile of serum copper/zinc ratio (*p* < 0.001). The multivariate hazard ratio (HR) for breast cancer death was 2.07 (95% CI 1.17–3.63; *p* = 0.01) for patients in the highest quartile of serum copper/zinc ratio compared to those in the lowest. There is evidence that the serum zinc level and copper/zinc ratio provide an independent predictive value for overall survival and breast cancer-specific survival after breast cancer diagnosis.

## 1. Introduction

Several groups have studied the influence of serum levels of trace elements on the survival of breast cancer patients [1,2,3]. We have previously reported that low serum selenium levels are associated with a decrease in 5-year and 10-year survival in breast cancer patients [4,5]. Metals of interest in breast cancer outcomes include zinc and copper. Zinc is involved in many physiological processes, including maintaining genomic stability, apoptosis, response to oxidative stress, and cell signaling [6,7,8]. Zinc is important for normal mammary gland growth and remodeling [9]. Zinc is required for effective T-cell functioning and thereby plays a role in anti-tumor immunity [10,11]. Serum zinc levels reflect dietary intake and supplement use [12]. Zinc blood levels have been evaluated as a possible risk factor for breast cancer [13]. In patients with breast cancer, the level of zinc (in serum, hair and tumor tissue) has been assessed in case-control studies [14,15,16,17] and in cohort studies [18,19,20,21,22,23,24,25,26,27,28,29,30,31,32,33,34]. In general, the case-control studies reported lower plasma zinc levels in breast cancer patients compared to healthy controls [15,16,17]. This suggests that zinc may be a biomarker of the presence of breast cancer rather than a risk factor. Copper plays a role in angiogenesis, tumor growth, cancer progression and metastasis [35]. Dysregulation of copper can result in the overproduction of reactive oxygen species. These particles affect the process of carcinogenesis by damaging DNA and proteins [36]. Due to its high affinity for estrogen receptor α (ERα), copper can activate cell proliferation through an estrogen-regulated pathway. The ability of the metals to activate a chimeric receptor containing the hormone-binding domain of ER alpha suggests that their effects are mediated through the hormone-binding domain [37]. A recently described form of cell death is mediated by copper (cuproptosis), and this process might impact the tumor microenvironment [38]. Studies from Korea showed higher copper levels in cancer patients than in the control groups [39]. Only a few studies have investigated the relationship between serum copper concentration and the risk of breast cancer with mixed results. The optimal balance between copper and zinc levels plays a key role in the function of many enzymes. Imbalance between copper and zinc (increasing copper and lowering zinc) impairs the antioxidant activity of several enzymes [40]. Chronic oxidative stress may increase the risk of breast cancer and affect the early stages of carcinogenesis and progression [41,42]. The copper-to-zinc ratio (Cu/Zn ratio) is thought to be a more accurate prognostic factor than copper or zinc levels alone [43]. A recent meta-analysis concluded that an increased Cu/Zn ratio is associated with an increased risk of breast cancer [44]. Epidemiological studies assessing the relationship between high copper and low zinc levels and survival have shown relatively poor survival for various cancer types [45,46]. Only a few studies have focused on breast cancer patients. In a Swedish study, no relationship was found between serum zinc levels and prognosis for breast cancer [47]. One study assessed the relationship between the serum levels of copper, zinc and the Cu/Zn ratio and overall survival in a group of patients with breast cancer. No relationship between zinc and copper levels and survival was seen, but a high Cu/Zn ratio was associated with relatively poor survival [48]. The aim of the current analysis is to assess the impact of serum zinc and copper levels and the Cu/Zn ratio on 10-year overall survival and breast cancer-specific survival in breast cancer patients.

## 2. Materials and Methods

### 2.1. Study Population

The study population consisted of women with breast cancer diagnosed between 2008 and 2015 and treated at two hospitals associated with the Pomeranian Medical University in Szczecin, Poland. The inclusion criterion for the study was blood sampling before the start of treatment and within three months of the diagnosis of breast cancer. We excluded patients with a diagnosis of pure DCIS, with metastatic disease at diagnosis (stage IV cancer) or with a previous history of another cancer. Patients had a consultation at the Hereditary Cancer Center in Szczecin. Each patient signed a written consent for blood collection. A blood sample was collected and stored for research purposes. Patients were tested for Polish founder mutations in *BRCA1* (c.5263_5264insC; c.4035delA; c.181T>G) as described previously [49]. Clinical data were obtained during an interview with the patient and from medical records. Detailed information about the study group was described previously [5].

### 2.2. Ethical Approval and Informed Consent

The study was conducted in accordance with the Declaration of Helsinki and was approved by the Ethics Committee of the Pomeranian Medical University in Szczecin.

### 2.3. Analytical Procedures

During the outpatient clinic visit, an 8 mL sample of peripheral blood was collected. Patients were asked to fast for at least four hours prior to blood collection. Tubes were incubated at room temperature for a minimum of 30 min to facilitate clotting and then were centrifuged for 12 min. The serum was deep frozen at −80 °C. We used a NexION 350D inductively coupled plasma mass spectrometer (Perkin Elmer, Perkin Elmer, Shelton, CT, USA) to measure copper and zinc levels. The spectrometer was equipped with Universal Cell Technology (UCT). Copper ^65^Cu and zinc ^66^Zn isotopes were selected for determination by ICP-MS. KED mode with helium (Kinetic Energy Discrimination or KED) was used for the reduction of polyatomic interferences. Calibration standards were prepared from 10 µg/mL Multi-Element Calibration Standard 3 (Perkin Elmer) by diluting with blank reagent to the final concentrations of 300, 450, 600, 750, 1500 and 2500 µg/L for copper and zinc. Correlation coefficients for calibration curves were greater than 0.999. The analysis protocol assumed a 30-fold dilution of serum in the blank reagent. The blank reagent consisted of high purity water (>18 MΩ), TMAH (AlfaAesar, Kandel, Germany), Triton X-100 (PerkinElmer, Shelton, CT, USA), *n*-butanol (Merck, Darmstadt, Germany) and disodium EDTA (Sigma Aldrich, Steinheim, Germany). Rhodium was set as the internal standard. ClinChek^®®^ Serum Control Level I (Recipe, Munich, Germany) was used as a reference material. Detailed information about the analytical procedures were described previously [50].

### 2.4. Statistical Analysis

We measured copper, zinc and Cu/Zn ratio for each patient. For both copper and zinc, we assigned patients to quartiles of equal size based on the serum concentrations. We considered the highest quartile of zinc to be the reference quartile and the lowest quartile of copper to be the reference quartile. We obtained information on the date of death from the National Statistical Register as of 16 May 2023. Cause of death was obtained from patients’ medical records and from general physicians. The observation period included the period from the diagnosis of breast cancer to the date of death or until 16 May 2023. Overall and breast cancer-specific survival rates were calculated by the Kaplan–Meier method. We used the Cox regression analysis to estimate hazard ratios for survival (overall and breast cancer specific). We performed a univariate analysis to identify variables that were significant predictors of death. Variables with a *p*-value ≤ 0.1 in the univariate analysis were included in the multivariate model. In this analysis, we considered a *p*-value ≤ 0.05 to be statistically significant. The analysis was conducted using TIBCO Software Inc. (2017) (Palo Alto, CA, USA) and Statistica (data analysis software system), version 13 (StatSoft, Krakow, Poland; http://statistica.io, accessed on 10 November 2020).

## 3. Results

### 3.1. Mean Plasma Zinc and Copper Levels in the Study Group

We included 538 patients in the study. The characteristics of the study group are presented in Table 1. The youngest age of onset was 26 years and the oldest was 92 years of age (the median was 57.2 years). There were 140 patients diagnosed at ≤50 years of age (26.0%). The majority of cases were node negative (61.5%), and the majority had a tumor of less than 5 cm in size (93.2%). The estrogen receptor was positive in 70.3% of and progesterone receptor in 63.8% primary tumors. HER2 positive status was detected in 16.5% of patients. The majority of patients (66.2%) had a total mastectomy. Chemotherapy (neo-adjuvant or adjuvant) was given to 54.2% of the patients, 61.3% received radiotherapy and 95.5% with positive estrogen receptor status received hormone therapy. The serum zinc level did not correlate with any of the other prognostic factors. However, the serum copper level was higher in those with node-positive cancer than in those with node-negative cancer and was higher among those who received chemotherapy than those who did not (blood was taken prior to the initiation of chemotherapy) (Table 1).

The mean zinc level was 847.3 μg/L (range: 525.21–1498.14 μg/L). The mean copper level was 1152.0 μg/L (range: 658.44–2153.12 μg/L). The distributions of copper and zinc levels by quartile are presented in Table 2.

### 3.2. Cox Regression Models for Overall and Breast Cancer-Specific Survival

In the univariate analysis, the following factors were statistically significant predictors of both all-cause mortality and breast cancer-specific mortality: type of surgery (lumpectomy versus mastectomy), lymph node status, tumor size, serum zinc level (quartile 4 vs. 1), serum copper level (quartile 1 vs. 4), Cu/Zn ratio (quartile 1 vs. 4). Additionally, chemotherapy was a significant predictor of breast cancer-specific mortality (yes/no) (Table 3). These factors were then included in the multivariate analyses of all-cause mortality and breast cancer-specific mortality (Table 4).

### 3.3. Overall and Breast Cancer Specific Survival by Quartiles of Zinc

**Figure 1:**
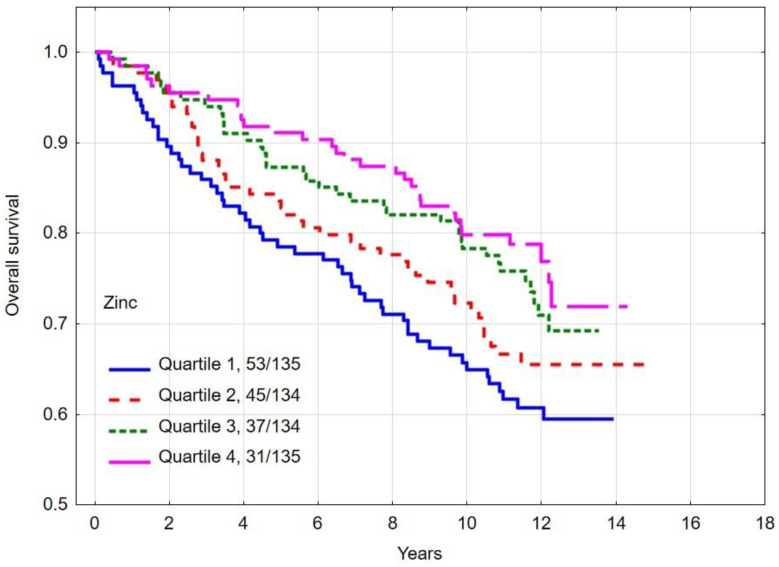
Ten-year all-cause mortality by quartiles of zinc levels.

**Figure 2:**
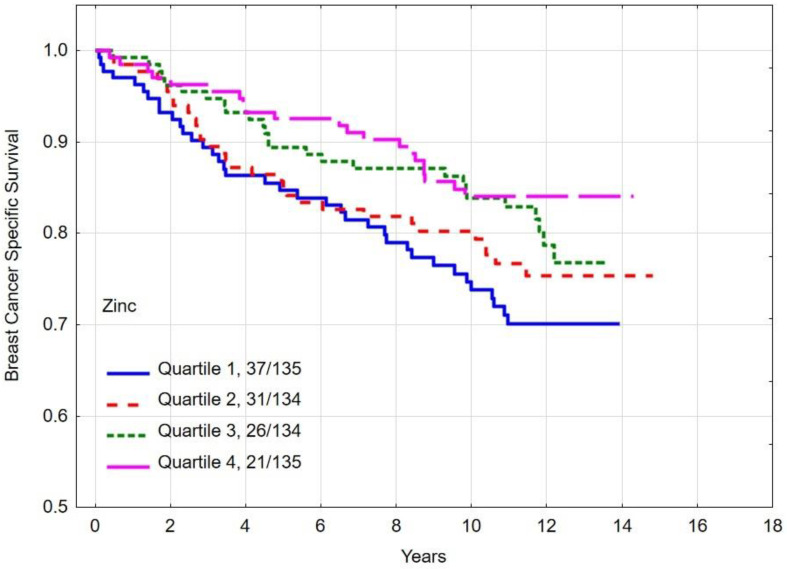
Ten-year breast cancer-specific survival by quartiles of zinc levels.

The multivariate hazard ratio (HR) for breast cancer-specific mortality was 1.85 (95% CI 1.02–3.37, *p* = 0.04) between women with the lowest (quartile 1) zinc levels and the highest (quartile 4). The absolute difference in 10-year overall survival between patients in the lowest and highest quartiles was 15.0% (*p*-long rank = 0.004). The 10-year overall survival rates and breast cancer specific survival rates by zinc level are presented in Table 5 and Figure 1 and Figure 2.

### 3.4. Overall and Breast Cancer-Specific Survival by Quartiles of Copper

**Figure 3:**
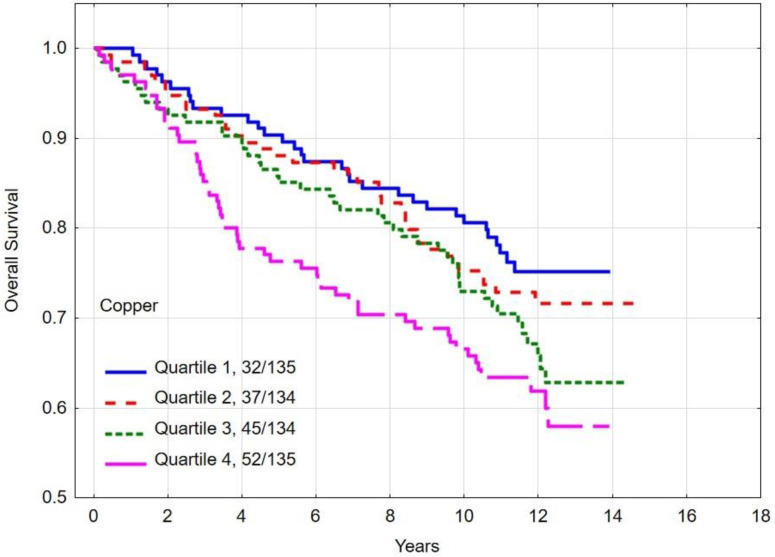
Ten-year all-cause mortality by quartiles of copper levels.

**Figure 4:**
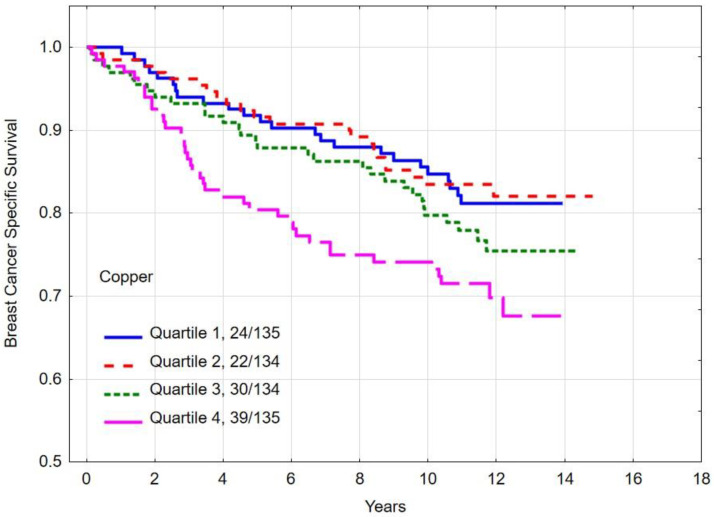
Ten-year breast cancer-specific survival by quartiles of copper levels.

The multivariate hazard ratio (HR) for breast cancer-specific mortality was 1.37 (95% CI 0.77–2.44, *p* = 0.28) (Table 4) between women with the highest (quartile 4) copper levels and the lowest (quartile 1). The absolute difference in 10-year overall survival between patients in the highest and lowest quartiles was 14.1% (*p*-long rank = 0.005). The 10-year overall survival rates and breast cancer survival rates by copper level are presented in Table 5 and Figure 3 and Figure 4.

### 3.5. Overall and Breast Cancer-Specific Survival by Quartiles of Copper/Zinc (Cu/Zn) Ratio

**Figure 5:**
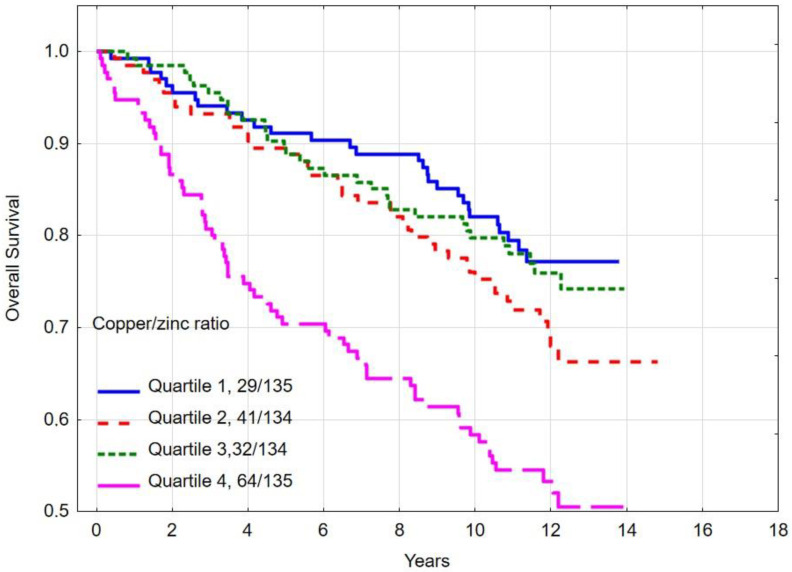
Ten-year all-cause mortality by copper/zinc (Cu/Zn) ratio quartile.

The 10-year overall survival rate was 58.3% for women in quartile 4 (highest levels) of the copper/zinc (Cu/Zn) ratio, 79.8% for quartile 3, 75.3% for quartile 2 and was 82.1% for quartile 1 (*p*-long rank < 0.001) (Table 5). Women in the highest copper/zinc (Cu/Zn) ratio level (quartile 4) compared to the lowest copper/zinc (Cu/Zn) ratio level (quartile 1) had a multivariate hazard ratio (HR) for all-cause mortality of 2.26 (95% CI 1.38–3.69, *p* = 0.0001) (Table 4). Overall survival by copper/zinc (Cu/Zn) ratio level is presented in Figure 5.

**Figure 6:**
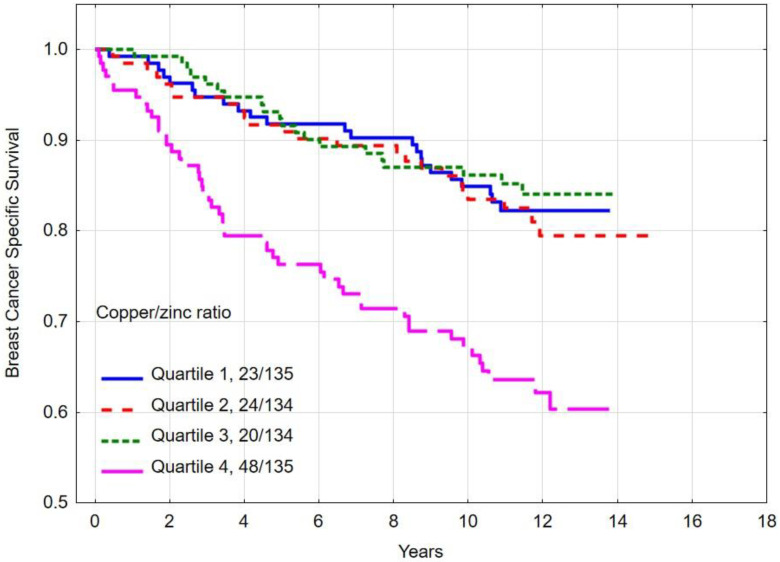
Ten-year breast cancer-specific survival by copper/zinc (Cu/Zn) ratio quartiles.

The 10-year breast cancer-specific survival rate was 67.2% for women in quartile 4 (highest level) of the copper/zinc (Cu/Zn) ratio level, 86.2% for quartile 3, 83.5% for quartile 2 and 84.9% for quartile 1 (*p*-long rank <0.001) (Table 5). Women with the highest copper/zinc (Cu/Zn) ratio level (quartile 4) compared to the lowest copper/zinc (Cu/Zn) ratio level (quartile 1) had a multivariate hazard ratio (HR) for breast cancer-specific mortality of 2.07 (95% CI 1.17–3.66, *p* = 0.01) (Table 4). Breast cancer-specific survival by copper/zinc (Cu/Zn) ratio is presented in Figure 6.

### 3.6. The Most Important Results

We present a summary table with all relevant results (Table 6).

## 4. Discussion

We have previously published survival data on this group of breast cancer patients according to serum selenium level, and now we present data on the relationship between survival and zinc, copper and cooper/zinc ratio levels [4,5]. In the present study, we observed that a low serum zinc level (≤762.7 μg/L) and high copper/zinc ratio (≥1.563) was associated with a decreased overall 10-year survival and breast cancer-specific survival. We observed a clear correlation between high copper/zinc ratio levels and overall survival (HR 2.26 95% CI 1.38–3.69; *p* = 0.0001) and breast cancer-specific survival (HR 2.07 95% CI 1.17–3.66; *p* = 0.01). The 10-year overall survival rate was 58.3% for women with the highest serum copper/zinc ratio quartile levels, compared to 83.1% among those in the lowest. The 10-year breast cancer-specific survival rate was 67.2% for women with the highest serum copper/zinc ratio quartile levels compared to 84.9% among those in the lowest quartile. Previously, the relationship between zinc levels and breast cancer risk was assessed by Bengtsson et al. in a group of 2372 breast cancer patients in a Swedish study. In that study, no associations were found between pre-diagnostic levels of serum zinc or dietary intake of zinc and breast cancer risk, and the authors determined that serum zinc is a poor marker of zinc intake [47]. However, in that study, blood samples for the assessment of serum zinc levels were collected between the years of 1991 to 1996 from healthy women. These women were then followed until December 31, 2019 for the diagnosis of breast cancer. Therefore, in many cases, several years passed from blood collection until the diagnosis of breast cancer, which may be the reason for the lack of correlation between zinc levels and survival. In our study, patients had blood drawn for testing within 90 days of the diagnosis of breast cancer. Last year, the authors of the previous study published a paper assessing the relationship between the levels of zinc, copper and the copper/zinc ratio on overall survival in breast cancer. The study did not find statistically significant relationship between zinc levels and overall survival, although there was a tendency toward lower breast cancer survival and lower zinc levels when comparing serum zinc Q2–Q4 vs. Q1 [48]. In our study, similarly to the Swedish study, we divided the patients into quartiles according to their zinc levels. However, in the Swedish study, the observation period was shorter because patients were included from 2010 and observed until 2019, and in our study, patients were included from 2008 and observed until May 2023, which may have affected the obtained results. It is necessary to obtain results from other research groups and populations to confirm the relationship between serum zinc levels and the survival of patients diagnosed with breast cancer. We observed a stronger relationship between the Cu/Zn ratio and overall survival and breast cancer-specific survival. Our results are consistent with the results of Bengtsson et al. in the Swedish study [48]. In our study, the difference in survival persisted during a long-term follow-up period. Previously, correlations between copper levels and the Cu/Zn ratio and survival in hepatocellular carcinoma were observed in a large prospective observational study in a group of 989 patients. Higher serum copper levels were strongly associated with worse cause-specific survival (Q4 vs. Q1: HR = 1.87, 95% CI: 1.22–2.86; *p* < 0.01 for trend) and overall survival (Q4 vs. Q1: HR = 2.06, 95% CI: 1.36–3.11; *p* < 0.01 for trend). There are several limitations of our study. There are many factors which influence prognosis, and these may impact serum zinc and/or copper levels. Possible confounders include family histories, dietary supplements, comorbidity, exercise and BMI. Due to the observational nature of our study, the data obtained should be confirmed in a prospective observation. The strength of this study is the long follow-up period with a median of almost 10 years. Another strength of the study is the inclusion of patients before starting breast cancer treatment and exclusion of patients during treatment. This is important because we did not have to consider the possible impact of breast cancer treatments (chemotherapy, hormone therapy, surgery, radiotherapy) on the serum levels of microelements. Literature data has described possible interactions between zinc and selenium, among others, in the process of activating the pathway of the mitogen-activated protein kinase pathway signaling proteins and activation of zinc finger proteins. It should be noted that selenium supplementation may affect the zinc status and consequent dysregulation of metallothionein synthesis [51]. Also, the interactions between zinc and copper can play a role in breast cancer development. However, the mechanism of breast cancer development is mainly related to the overall impact on oxidative stress and is correlated with tumorigenesis and cancer progress. The elevated Cu/Zn ratio is negatively correlated with antioxidants and positively correlated with oxidation products [52]. Zinc is recognized as an important cofactor for over 300 enzymes involved in inflammation, oxidation, immune response and cell death programs. Zinc is an essential component of copper-zinc dismutase (CuZnSOD) and approximately 1000 transcription factors. An imbalance between zinc and copper levels can lead to oxidative stress and impairment of the antioxidant function of many enzymes. The exact mechanism between copper and zinc and the development of breast cancer is still unknown. Recently, research has been published on the role of mitochondrial DNA in breast cancer risk and prognosis. Mutations and epigenetic modification in the mitochondrial genome facilitate breast cancer initiation and progression [53]. Mitochondrial DNA is sensitive to oxidative and other genotoxic damage. There is a high concentration of oxygen species in the mitochondria [54]. As we know, an imbalance between copper and zinc can result in the overproduction of reactive oxygen species. Mitochondrial DNA is particularly sensitive to these molecules, and there may be a theoretical link between copper and zinc levels and mutations in mitochondrial DNA in breast cancer cells. Additionally, recently published articles on cuproptosis showed the importance of mitochondria in this process. Cuproptosis is copper-dependent cell death. Intracellular copper concentrations are kept at extraordinarily low levels [55]. The mechanism of copper-induced cell death involves intracellular copper accumulation. Recent published data has shown that copper-dependent death occurs via direct binding of copper to lipoylated components of the tricarboxylic acid (TCA) cycle and is related to mitochondrial function [56]. We plan to assess the correlation between three elements (zinc, copper and selenium) and the prognosis of breast cancer in the future, but this requires increasing the size of the study group and probably extending the observation period. Finally, our results suggest the potential for zinc supplementation in breast cancer patients with low zinc levels. So far, zinc supplementation has been tested in a small study in a group of patients with colorectal cancer undergoing adjuvant chemotherapy. In that study, zinc supplementation during chemotherapy cycles increased antioxidant enzymes superoxide dismutase activity and maintained vitamin E concentrations [57]. There are currently no clinical trials on zinc supplementation in patients with breast cancer. Testing this hypothesis in breast cancer patients requires planning interventional studies with random selection in subgroups.

## 5. Conclusions

We conclude that a high copper/zinc ratio (≥1.563) at the time of a breast cancer diagnosis is associated with decreased overall 10-year survival and breast cancer-specific survival.

## Figures and Tables

**Table 1:** Study population and mean plasma zinc and copper levels by various features.

Risk Factor		*n*	%	Mean Zinc Level [µg/L]	SD	*p*	Mean Copper Level [µg/L]	SD	*p*
All		538	100	847.3	±129.2		1152.0	±217.6	
Age range (mean, SD)		25–92 (57.2 ± 12.1)	n/a						
	≤50	140	26.0	854.4	±125.3	0.45	1143.3	±263.1	0.58
	>50	398	74.0	844.8	±130.6		1155.1	±199.4	
*BRCA1* mutation									
	Yes	62	11.5	840.1	±115.6	0.64	1138.1	±185.9	0.59
	No	476	88.5	848.3	±130.9		1153.9	±221.5	
Tumor size [cm]									
	≤2.0	316	58.8	846.9	±127.3	0.40	1142.1	±226.2	0.26
	2.1–5.0	185	34.4	855.8	±129.1		1161.1	±197.4	
	≥5.1	12	2.2	807.7	±131.2		1234.3	±297.4	
	Missing	25	4.6	808.5	±149.2		1170.9	±206.8	
Lymph node status									
	Positive	194	36.1	847.1	±122.0	0.79	1190.8	±226.0	0.002
	Negative	331	61.5	850.3	±133.2		1130.5	±211.2	
	Missing	13	2.4	774.9	±115.2		1123.3	±178.1	
ER status									
	Positive	378	70.3	844.1	±129.6	0.26	1142.7	±212.1	0.11
	Negative	152	28.3	858.0	±126.9		1176.2	±231.7	
	Missing	8	1.4	797.2	±150.8		1131.5	±183.6	
PR status									
	Positive	343	63.8	844.2	±132.0	0.33	1142.8	±212.2	0.14
	Negative	174	32.3	856.0	±124.9		1173.1	±229.2	
	Missing	21	3.9	825.5	±118.6		1128.5	±200.9	
HER2 status									
	Positive	89	16.5	864.7	±146.6	0.20	1148.4	±219.9	0.78
	Negative	423	78.7	845.5	±125.7		1155.5	±218.3	
	Missing	26	4.8	817.8	±118.7		1107.8	±199.4	
Hormone therapy; ER(+)									
	Yes	361	95.5	845.6	±128.8	0.29	1145.1	±211.5	0.32
	No	17	4.5	811.9	±145.9		1092.9	±224.0	
Radiotherapy									
	Yes	330	61.3	852.1	±130.2	0.28	1149.7	±214.6	0.74
	No	193	35.9	839.5	±126.2		1156.1	±224.4	
	Missing	15	2.8	842.5	±149.8		1151.6	±206.3	
Chemotherapy									
	Yes	292	54.2	849.6	±126.9	0.86	1169.5	±232.9	0.03
	No	230	42.8	847.7	±131.9		1128.7	±197.5	
	Missing	16	3.0	799.6	±130.5		1167.4	±179.9	
Type of surgery									
	Mastectomy	356	66.2	846.8	±123.2	0.39	1158.8	±220.7	0.14
	Lumpectomy	163	30.3	857.2	±137.5		1128.5	±208.3	
	Missing	19	3.5	772.5	±147.3		1228.1	±220.6	
Vital status									
	Alive	372	69.1	860.4	±130.9		1139.4	±214.9	
	Dead	166	30.9	818.0	±120.7	<0.001	1180.3	±221.5	0.04
	Dead of breast cancer	115	21.4	815.5	±116.4	0.003	1187.9	±239.7	0.05
	Dead of other cancers	13	2.4	826.8	±105.7	0.56	1122.5	±222.0	0.62
	Dead of any cancers	128	23.8	816.7	±115.1	0.002	1181.3	±237.9	0.08
Smoking status									
	Yes, current	116	21.6	847.8	±126.8	0.26	1188.5	±237.4	0.05
	Yes, past	140	26.0	861.3	±135.6		1155.0	±231.1	
	Never	273	50.7	839.6	±123.0		1130.6	±196.5	
	Missing	9	1.7	860.0	±223.5		1287.3	±265.6	

**Table 2:** Distribution of zinc and copper levels in entire sample.

Quartile	Number of Patients	Zinc μg/L	Copper μg/L	Cu/Zn Ratio
1	135	525.2–762.7	658.4–1012.4	0.769–1.160
2	134	762.8–839.7	1012.7–1122.3	1.161–1.321
3	134	840.2–919.6	1122.8–1254.6	1.322–1.562
4	135	920.3–1498.1	1255.3–2153.1	1.563–3.003

**Table 3:** Cox regression models for overall and breast cancer-specific survival: univariate analysis.

Risk Factor		All-Cause Mortality			Breast Cancer-Specific Mortality		
		HR (95% CI)		*p*	HR (95% CI)		*p*
Age							
	≤50	1.00	Reference		1.00	Reference	
	≥51	1.33	(0.91–1.93)	0.14	0.96	(0.64–1.45)	0.86
*BRCA1* mutation							
	No	1.00	Reference		1.00	Reference	
	Yes	1.18	(0.71–1.94)	0.52	0.70	(0.36–1.39)	0.31
Lymph node status							
	Negative	1.00	Reference		1.00	Reference	
	Positive	2.56	(1.87–3.51)	<0.001	3.51	(2.38–5.18)	<0.001
ER status							
	Negative	1.00	Reference		1.00	Reference	
	Positive	1.18	(0.82–1.69)	0.37	1.11	(0.74–1.67)	0.93
PR status							
	Negative	1.00	Reference		1.00	Reference	
	Positive	1.28	(0.90–1.83)	0.17	0.98	(0.65–1.48)	0.93
HER2 status							
	Negative	1.00	Reference		1.00	Reference	
	Positive	1.06	(0.70–1.60)	0.79	0.85	(0.52–1.39)	0.53
Tumor size [cm]							
	0–1.9	1.00	Reference		1.00	Reference	
	2.0–4.9	1.94	(1.38–2.71)	<0.001	2.30	(1.51–3.47)	<0.001
	≥5.0	6.14	(3.15–11.9)	<0.001	7.75	(3.62–16.6)	<0.001
Radiotherapy							
	No	1.00	Reference		1.00	Reference	
	Yes	0.95	(0.68–1.32)	0.40	0.84	(0.56–1.27)	0.42
Chemotherapy							
	No	1.00	Reference		1.00	Reference	
	Yes	1.27	(0.91–1.96)	0.15	1.78	(1.17–2.67)	0.007
Type of surgery							
	Lumpectomy	1.00	Reference		1.00	Reference	
	Mastectomy	1.99	(1.34–2.96)	<0.001	2.24	(1.36–3.70)	0.001
Hormone therapy (only ER positive)							
	No	1.00	Reference		1.00	Reference	
	Yes	1.03	(0.42–2.53)	0.94	1.22	(0.44–3.33)	0.70
Smoking							
	Never	1.00	Reference		1.00	Reference	
	Yes, past	0.82	(0.56–1.19)	0.52	0.77	(0.49–1.21)	0.26
	Yes, current	0.88	(0.59–1.30)	0.88	0.76	(0.42–1.16)	0.17
Zinc quartile							
	Quartile 4	1.00	Reference		1.00	Reference	
	Quartile 1	1.91	(1.23–2.98)	0.004	1.98	(1.16–3.38)	0.01
	Quartile 2	1.56	(0.99–2.47)	0.06	1.59	(0.91–2.76)	0.10
	Quartile 3	1.20	(0.74–1.93)	0.46	1.25	(0.70–2.22)	0.45
Copper quartile							
	Quartile 1	1.00	Reference		1.00	Reference	
	Quartile 2	1.18	(0.73–1.89)	0.50	0.94	(0.52–1.67)	0.82
	Quartile 3	1.47	(0.93–2.31)	0.10	1.31	(0.76–2.24)	0.33
	Quartile 4	1.87	(1.20–2.90)	0.005	1.85	(1.11–3.08)	0.02
Copper/Zinc ratio quartile							
	Quartile 1	1.00	Reference		1.00	Reference	
	Quartile 2	1.46	(0.91–2.35)	0.12	1.08	(0.61–1.91)	0.79
	Quartile 3	1.10	(0.67–1.82)	0.70	0.87	(0.48–1.59)	0.66
	Quartile 4	2.72	(1.75–4.21)	<0.001	2.55	(1.55–4.19)	<0.001

**Table 4:** Cox regression models for overall and breast cancer-specific survival: multivariate analysis.

Risk Factor			All-Cause Mortality				Breast Cancer-Specific Mortality			
		At Risk (n)	Events (n)	HR (95% CI)		*p* ^a^	Events (n)	HR (95% CI)		*p* ^b^
Zinc quartile										
	Quartile 4	135	31	1.00	Reference		21	1.00	Reference	
	Quartile 1	134	53	1.74	(1.07–2.81)	0.02	37	1.85	(1.02–3.37)	0.04
	Quartile 2	134	45	1.48	(0.91–2.41)	0.11	31	1.512	(0.83–2.78)	0.18
	Quartile 3	135	37	1.12	(0.68–1.85)	0.65	26	1.11	(0.60–2.07)	0.74
Copper quartile										
	Quartile 1	135	32	1.00	Reference		24	1.00	Reference	
	Quartile 2	134	37	1.15	(0.70–1.91)	0.58	22	0.97	(0.52–1.80)	0.91
	Quartile 3	134	45	1.34	(0.82–2.18)	0.23	30	1.14	(0.63–2.06)	0.67
	Quartile 4	135	52	1.49	(0.92–2.42)	0.10	39	1.37	(0.77–2.44)	0.28
Copper/zinc ratio quartile										
	Quartile 1	135	29	1.00	Reference		23	1.00	Reference	
	Quartile 2	134	41	1.56	(0.93–2.62)	0.09	24	1.18	(0.63–2.20)	0.61
	Quartile 3	134	32	1.37	(0.80–2.34)	0.25	20	1.07	(0.56–2.07)	0.83
	Quartile 4	135	64	2.26	(1.38–3.69)	0.001	48	2.07	(1.17–3.66)	0.01

^a^ Mutually adjusted for variables: lymph node status, tumor size, type of surgery; ^b^ Mutually adjusted for variables: lymph node status, tumor size, chemotherapy, type of surgery.

**Table 5:** Ten-year overall and breast cancer-specific survival by quartiles of zinc, copper and copper/zinc ratio.

Risk Factor		Overall Survival (OS)		Breast Cancer-Specific Survival	
		10 Year (%)	Log-Rank Test	10 Year (%)	Log-Rank Test
All		73.9	*p*	80.5	*p*
Zinc quartile					
	Quartile 1	64.9	0.006 ^a^	73.8	0.04 ^b^
	Quartile 2	72.2		80.3	
	Quartile 3	78.2		83.7	
	Quartile 4	79.9		84.3	
Zinc quartile					
	Quartile 2–4	76.8	0.01	82.7	0.03
	Quartile 1	64.9		73.8	
Copper quartile					
	Quartile 1	80.6	0.03 ^c^	84.8	0.02 ^d^
	Quartile 2	75.2		83.4	
	Quartile 3	72.9		79.7	
	Quartile 4	66.5		74.1	
Copper quartile					
	Quartile 1–3	76.3	0.01	82.7	0.007
	Quartile 4	66.5		74.1	
Copper/zinc ratio quartile					
	Quartile 1	82.1	<0.001 ^e^	84.9	<0.001 ^f^
	Quartile 2	75.3		83.5	
	Quartile 3	79.8		86.2	
	Quartile 4	58.3		67.2	
Copper/zinc ratio quartile					
	Quartile 1–3	79.0	<0.001	84.9	<0.001
	Quartile 4	58.3		67.2	

^a^ zinc quartile 1 vs. 4 (*p* = 0.004); 1 vs. 3 (*p* = 0.03); 1 vs. 2 (*p* = 0.30); ^b^ zinc quartile 1 vs. 4 (*p* = 0.009); 1 vs. 3 (*p* = 0.07); 1 vs. 2 (*p* = 0.36); ^c^ copper quartile 4 vs. 1 (*p* = 0.005); 4 vs. 2 (*p* = 0.03); 4 vs. 3 (*p* = 0.24); ^d^ copper quartile 4 vs. 1 (*p* = 0.02); 4 vs. 2 (*p* = 0.01); 4 vs. 3 (*p* = 0.15); ^e^ copper/zinc ratio quartile 4 vs. 1 (*p* < 0.001); 4 vs. 2 (*p* = 0.002); 4 vs. 3 (*p* < 0.001); ^f^ copper/zinc ratio quartile 4 vs. 1 (*p* < 0.001); 4 vs. 2 (*p* < 0.001); 4 vs. 3 (*p* < 0.001).

**Table 6:** Summary table.

Risk Factor		All-Cause Mortality					Breast Cancer-Specific Mortality				
		HR (95% CI) Multivariate Analysis		*p*	10-Year Survival (%)	*p*	HR (95% CI) Multivariate Analysis		*p*	10-Year Survival (%)	*p*
Zinc quartile											
	Quartile 4	1.00	Reference		79.9	0.004	1.00	Reference		84.3	0.009
	Quartile 1	1.74	(1.07–2.81)	0.02	64.9		1.85	(1.02–3.37)	0.04	73.8	
Copper/zinc ratio quartile											
	Quartile 1	1.00	Reference		82.1	<0.001	1.00	Reference		84.9	<0.001
	Quartile 4	2.26	(1.38–3.69)	0.001	58.3		2.07	(1.17–3.66)	0.01	67.2	

## Data Availability

The data presented in this study are available on request from the corresponding author due to the source data are materials for subsequent publications in the habilitation process.

## Abbreviations

BRCA1: breast cancer 1 gene; Cu: copper; DCIS: ductal carcinoma in situ; CI: confidence interval; ER: estrogen receptor; HR: hazard ratio; LCSS: liver cancer-specific survival; OS: overall survival; PR: progesterone receptor; Zn: zinc.
